# Opportunities and Challenges for Predicting the Service Status of SLM Metal Parts Under Big Data and Artificial Intelligence

**DOI:** 10.3390/ma17225648

**Published:** 2024-11-19

**Authors:** Xiaoling Yan, Huiwen Fu

**Affiliations:** School of Computer and Artificial Intelligence, Beijing Technology and Business University, Beijing 102488, China; fhw0706@163.com

**Keywords:** nonlinear ultrasonic wave, big data, artificial intelligence, SLM metal parts, service status

## Abstract

Selective laser melting (SLM) technology is a high-end dual-use technology that is implemented in aerospace and medical equipment, as well as the automotive industry and other military and civilian industries, and is urgently needed for major equipment manufacturing and national defense industries. This paper examines the challenges of uncontrollable service states and the inability to ensure service safety of SLM metal parts under nonlinear and complex operating conditions. An overview of the prediction of the service status of SLM metal parts was introduced, and an effective approach solving the problem was provided in this paper. In this approach, the cross-scale coupling mechanism between mesoscopic damage evolution and macroscopic service state evolution is clarified by tracking the mesoscopic damage evolution process of SLM metal parts based on ultrasonic nonlinear responses. The failure mechanism is organically integrated with hidden information from monitoring big data, and a “chimeric” model to accurately evaluate the service status of SLM metal parts is constructed. Combining nonlinear ultrasound technology with big data and artificial intelligence to construct a “chimeric” model and consummate the corresponding methods and theories for evaluating the service status of SLM metal parts is an effective way to reveal the mesoscopic damage evolution and service status evolution mechanisms of SLM metal parts under complex factor coupling, and to accurately describe and characterize the service status of parts under complex operating conditions. The proposed approach will provide a theoretical basis and technical guarantee for the precise management of SLM parts’ service safety in key equipment fields such as aerospace, medical equipment, and the automotive industry.

## 1. Introduction

**Figure 1:**
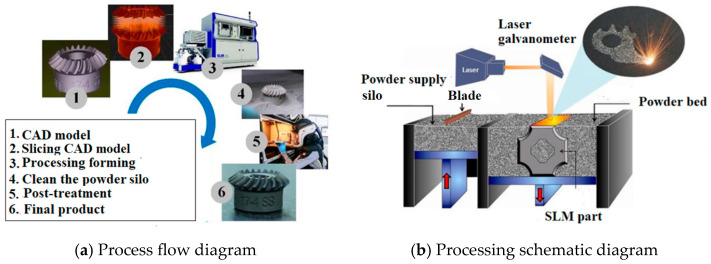
Basic flow and processing schematic of SLM process.

SLM is considered to be one of the most promising additive manufacturing technologies that is capable of forming functional parts with almost 100% density of any complex structure. It is a high-end technology that can be used for both military and civilian purposes in aerospace, medical equipment, automotive industry, and other industries [1,2,3]. SLM is urgently needed for major equipment manufacturing and the defense industry and is a key technology of strategic importance to the country. As shown in Figure 1, when a part is manufactured by SLM technology, a high-energy laser beam is used as a moving heat source, and the pre-laid metal powder is rapidly heated, melted, and solidified to form a “micro melt pool” unit. Along the moving direction of the laser beam, the “micro-molten pool” units are continuously accumulated to form melt paths with different molten widths and residual heights, and these melt channels gather in a certain arrangement to form a plane. Then, multiple layers of undulating planes accumulate in the height direction of the formed part to form a three-dimensional part. As can be seen, the SLM forming process is an additive manufacturing process that spans multiple spatial scales and involves numerous influencing factors and multiple physical processes. Therefore, some microscopic defects [3,4,5,6] (inclusions, pores, cracks, etc.) are inevitably generated in SLM parts and are difficult to eliminate completely by post-treatment. Components subjected to long-term service processes, under the influence of fatigue loading and corrosive media, will experience performance degradation. For example, in July 2017, the U.S. Marine Corps KC-130 transport plane crash resulted in the death of all 16 military personnel on board; the accident investigation committee ultimately found that the ground crew responsible for maintenance did not carefully inspect and properly handle the corrosion of the engine propeller blade, which ultimately led to the tragic accident [4]. Therefore, it is of great significance to adopt reliable technology for the early detection of parts damage, accurately assess their service status, and implement personalized and precise health management according to the assessment results to effectively guarantee the operational safety, reliability, and economy of important parts.

Both theoretical studies and practical experience [2,3,4,5,6,7] have shown that the macroscopic damage and failure behavior of SLM metal parts originates from the mesoscopic scale deformation and damage evolution within the parts. Capturing the evolution of discrete dislocations, pores, microcracks, and other defects in SLM metallic materials at mesoscopic scales is of great theoretical significance for revealing the physical mechanisms of failure and service life evolution of SLM metallic parts. However, even for widely used iron-based alloy selective laser melting materials, only an empirical understanding of mesoscopic damage evolution and service state evolution has been accumulated [8,9,10,11,12,13]. Theories that quantitatively reveal the service state evolution laws of SLM parts, based on mesoscopic-scale damage evolution characteristics, have not yet been established. In recent years, with the development of technologies such as sensors, the Internet of Things (IoT), and 5G communications, the types and quantities of data collected in part service safety monitoring systems have increased dramatically, and as storage costs have been reduced, more and more data are being stored, resulting in the formation of “monitoring big data”. The “big data” collected and stored by the monitoring system during actual operation is a “sleeping treasure”, and the ability to fully utilize its implicit characteristics is the key to clarifying the characteristics of the monitoring system in different practical applications.

Non-destructive testing (NDT) is an effective means of evaluating internal damage in SLM metal parts. Conventional NDT methods [14,15,16,17,18,19,20], such as ultrasonic testing, eddy current testing, X-ray testing, penetration testing, and magnetic particle testing, are effective in detecting macro-structural defects such as cracks, porosity, and inclusions, but they are ineffective in detecting early damage. An ideal system for evaluating the in-service condition of a part should be able to detect damage at its earliest stages and accurately predict its remaining life, thus providing an assessment of the part’s safety. Nonlinear ultrasonic testing [21,22,23] has the advantage of being non-destructive, fast, with a large detection range, etc., and can essentially reflect the influence of small defects within the material on the ultrasonic propagation process, even if it is a small amount of damage (micron level). Ultrasonic propagation in the material will appear, such as waveform distortion, high harmonic breeding, the formation of sidebands, and other obvious nonlinear phenomena. In recent years, evaluating the degree of damage (fatigue, aging, creep, etc.) in metallic materials based on nonlinear ultrasound [24,25,26] has become a hot research topic, and many valuable research results have been obtained. Therefore, based on ultrasound nonlinear response tracking of the mesoscopic damage evolution process of SLM metal parts, the cross-scale coupling mechanism between mesoscopic damage evolution and macroscopic service state evolution is clarified. By using “monitoring big data” to further fully express the “personalized” implicit characteristics of the parts, an intelligent evaluation “chimeric” model for the service state of SLM metal parts is constructed. While revealing the universal scientific principles of SLM metal part failure, it can be matched with complex practical situations. Applying the “chimeric” model to accurately evaluate the service status of SLM parts, taking into account the economic and environmental benefits, implementing personalized and precise health management of the parts at the “precise timing” of the evolution of SLM part service status (health status, quantified performance degradation status (remaining life estimation), and failure status), fundamentally solving the problem of uncontrollable service status and inability to guarantee service safety of SLM metal parts under nonlinear and complex conditions, has great theoretical research and practical application significance.

In recent years, with the development and application of intelligent manufacturing technology, SLM metal parts have been increasingly used in aerospace, medical equipment, the automotive industry, and other fields. How to ensure the service safety of SLM metal parts under nonlinear and complex operating conditions is a key issue that urgently needs to be solved. At present, many scholars have applied machine learning, statistical data-driven methods, and other techniques to the intelligent evaluation of the service status of electromechanical equipment. However, although the existing methods can solve some problems to some extent, the limitations are also obvious. For example, machine learning cannot quantify the uncertainty of service status prediction; statistical data-driven methods have a limited ability to process big data; and the mechanism model + data-driven model methods cannot solve the errors introduced by unreasonable model combinations. How, then, can we fundamentally solve the problem of the uncontrollable service status of SLM metal parts under nonlinear and complex operating conditions? The research method proposed in this paper has great theoretical research value and practical application significance, as it is a key problem that urgently needs to be solved in fields such as aerospace, medical equipment, and the automotive industry.

## 2. Research Progress and Development Trend

SLM additive manufacturing technology has been a hot research topic, both domestically and internationally, in recent years, but the main research directions focus on powder preparation, forming processes, and forming equipment [27,28,29,30]. Research on the service safety and life prediction of SLM metal parts is not deep enough. Walker et al. [31] studied the effect of defects such as porosity and incomplete fusion on the fatigue life of Ti-6Al-4V specimens formed by SLM. The results showed that under ultra-high-cycle fatigue loading, inclusions and pores inside the specimens led to crack initiation and propagation, but there was no quantitative analysis of the effect of inclusions and pores defects on the fatigue life of SLM specimens. Bao et al. [32] obtained the geometric characteristics of fatigue defects in Ti-6Al-4V specimens prepared by SLM through synchrotron X-ray tomography and used machine learning to study the qualitative effects of defect location, size, and morphology on the fatigue life of the specimens. Chen et al. [33] conducted a series of low-cycle fatigue tastings on SLM 316L stainless steel specimens with different strain amplitudes, and the results showed that the life prediction method based on the non-Masing model is applicable to SLM 316L stainless steel specimens. As can be seen, current research on the service safety life cycle of SLM metal parts has only accumulated an empirical understanding of the relationship between mesoscopic damage evolution and service life evolution, and the research is still at levels of “experience” and “ambiguity”.

By using conventional methods such as fatigue loading experiments and microstructure analysis to understand the mesoscopic damage evolution state of SLM parts, a failure mechanism model is established [34,35], and the remaining life of the parts is predicted using the failure mechanism model. Based on this, evaluating the service status of the parts and achieving personalized health management is a classic method to ensure the safety of a part’s service. However, due to the complexity of the SLM component structure and the diversity of operating environments in practical applications, the evolution law of its service state is often difficult to physically model, or the cost of obtaining failure mechanism models is too high. This method is obviously not suitable for widespread application.

To monitor the mesoscopic damage evolution process of SLM parts using nonlinear ultrasound methods, it is necessary to establish a mapping relationship between the mesoscopic damage evolution and ultrasonic nonlinear response. From this perspective, current research on the mesoscopic damage monitoring of metal parts based on nonlinear ultrasound can be divided into three aspects.

### 2.1. Research on Nonlinear Ultrasound Theory

#### 2.1.1. Classical Nonlinearity Theory

The sources of acoustic nonlinearity in solid media can be divided into two categories: classical nonlinearity and contact acoustic nonlinearity. The classical nonlinear theory based on the stress–strain relationship [36,37,38] indicates that the non-harmonic nature of the material lattice and crystal defects are the main sources of ultrasonic nonlinearity, mainly manifested by the generation of harmonic components of the excitation sound wave. Cantrell [39] established the nonlinear wave equation for solid media based on the discrete lattice model, while Breazeale et al. [40] derived the one-dimensional longitudinal wave nonlinear wave equation based on the continuous medium model. Solving the nonlinear wave equation can obtain the relationship between harmonic amplitude and nonlinear coefficients. The approximate solution of the wave equation [40] for continuous sine harmonic excitation is

(1) $$u(x,t)=A_{1}\sin(kx-\omega t)+\frac{\beta A_{1}^{2}k^{2}x}{8}\cos(2kx-2\omega t)$$

where *ω* is the angular frequency of the excited ultrasonic wave, *x* is the propagation distance of the ultrasonic wave, *t* is the time, *k* is the wave number, and *A*_1_ is the amplitude of the fundamental wave and the amplitude of the second harmonic of the received ultrasonic wave is *A*_2_ = (*A*_1_^2^*k*^2^*x*)/8. The nonlinear coefficient *β* can be expressed as

(2) $$\beta =8(\frac{A_{2}}{A_{1}^{2}})\frac{1}{k^{2}x}$$

As can be seen, *β*∝ *A*_2_/*A*_1_^2^, *β* can characterize the mesoscopic damage evolution of materials but can only qualitatively describe the nonlinearity of materials from a macroscopic perspective. To achieve a quantitative evaluation of material damage, effective physical models must be developed. Thus, a quantitative relationship between the material mechanical performance parameters and ultrasonic nonlinear characteristics needs to be established. The current research mainly starts from the microstructure of materials, exploring the relationship between “material microstructure-material macroscopic mechanical properties-ultrasonic nonlinear behavior”. At present, research in this area mainly focuses on metal materials and two physical models have been proposed: the dislocation monopole model and the dislocation pair model.

Granato [41] developed a pinned dislocation monopole model for the interaction between ultrasound and dislocations. The relevant research results show that the generation of a second-harmonic ultrasound depends on the displacement motion of a single dislocation between the pinned points under stress, and the theoretical formula can be expressed as

(3) $$A_{2}=\frac{1}{4}[(\frac{E_{2}}{E_{1}}+\frac{12}{5}\frac{E_{1}^{2}\Omega \Lambda L^{4}R^{3}}{\mu^{3}b^{2}}\sigma ){\left(A_{1}k\right)}^{2}\frac{e^{-2\alpha_{1}x}-e^{-\alpha_{2}x}}{\alpha_{2}-\alpha_{1}}$$

where *E*_1_ and *E*_2_ are the second-order and third-order elastic constants, Λ is the dislocation density, *L* is the dislocation line length, *R* is the conversion coefficient of shear stress and normal stress, *μ* is shear modulus, *σ* is the normal stress, *b* is the Burgers vector, *α*_1_ and *α*_2_ are the fundamental wave attenuation and second-harmonic attenuation, and Ω is the conversion coefficient between tangential strain and normal strain.

With the deepening degradation of material properties, the dislocation structure is not only an arrangement of isolated dislocations but also includes a complex distribution of dislocation dipoles and multipoles. Based on this theory, Cantrell et al. [42] proposed a new model of second-harmonic excitation by ultrasound interacting with dislocation dipoles and dipole arrays, which suggests that at high dislocation densities, the ultrasonic second-harmonic amplitude is not only related to the length of the dislocation line and stress but also depends largely on the arrangement of dislocations in the structure. The theoretical formula can be expressed as

(4) $$\beta =\frac{16\pi^{2}\Omega \Lambda R^{2}h^{3}{\left(1-\nu \right)}^{2}E_{1}^{2}}{\mu^{2}b}+\frac{384\pi^{3}\Omega \Lambda R^{3}h^{4}{\left(1-\nu \right)}^{3}E_{1}^{2}}{\mu^{3}b^{2}}\sigma$$

where *h* is the distance of the dislocation dipole, and *ν* is Poisson’s ratio. In general, significant progress has been made in the study of classical nonlinear ultrasound theory, but further improvement is still needed, such as the lack of physical models for amorphous structures.

#### 2.1.2. Contact Acoustic Nonlinearity Theory

Contact acoustic nonlinearity theory indicates that when ultrasonic waves act on contact–type damages such as cracks and delamination, the nonlinear response of ultrasonic waves comes from two aspects: firstly, the elastic asymmetry of the contact interface under tension–compression action leads to the dynamic nonlinearity of an ultrasonic response. Secondly, the hysteresis effect of an ultrasonic response is caused by the transition of the opening–closing state of the damaged interface. The contact nonlinear theoretical models proposed by domestic and foreign scholars can be divided into two categories. One is the physical model, which studies the influence of contact-type damage on ultrasonic responses from the microscopic physical mechanism of materials. For example, Pecorari et al. [43,44] analyzed the influence of crack contact interface roughness on ultrasonic wave propagation, established the correlation between second-harmonic and interface roughness, and qualitatively explained the hysteresis effect caused by the rough contact interface. The other category is phenomenological modeling, in which an equivalent model is used to characterize the contact-like damage evolution process, which is then combined with the ultrasonic dynamic equations to analyze the relationship between the ultrasonic nonlinear response and material damage. The P–M (Presaich and Mayergoyz) spatial model proposed by McCall and Guyer [45] is the most widely used, which suggests that the nonlinearity of damaged materials is manifested at the microscopic level as a step response of particle stress–strain and at the mesoscopic level, as a hysteresis effect of solid materials, with many mesoscopic units forming macroscopic nonlinearity. By combining the P–M spatial model with the ultrasonic wave equation, the relationship between the ultrasonic response characteristics and nonlinear coefficients can be obtained, and the degree of material damage can be evaluated based on the relationship between nonlinear coefficients and distribution parameters.

In summary, significant progress has been made in the research of nonlinear ultrasound theory, both domestically and internationally. However, existing models such as dislocation monopoles, dislocation dipoles, and P–M space models are unable to independently characterize the entire life cycle of the damage evolution process of metal parts. Therefore, it is necessary to strengthen the basic research on nonlinear ultrasonic technology, consider the effects of attenuation, noise, and other factors, and establish a physical model more suitable for the actual system to achieve a leap from qualitative to quantitative evaluations of material damage based on nonlinear ultrasonic responses, and from qualitative interpretations to mechanism analysis.

### 2.2. Numerical Simulation Study of Various Nonlinear Acoustic Phenomena in Solids

**Figure 2:**
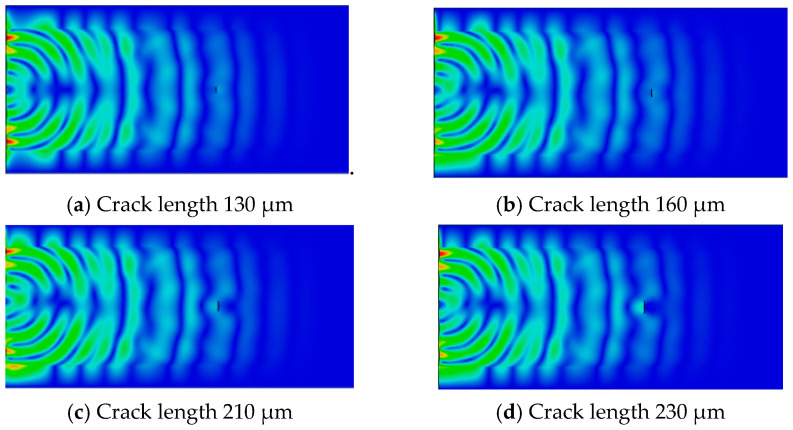
Transient diagram of ultrasonic wave field propagation at different stages of microcrack length propagation in SLM 316L stainless steel.

Due to the extremely complex propagation of ultrasonic waves in metal parts and their interaction with mesoscopic damage, it is difficult to establish an accurate mechanism model. Therefore, combining the nonlinear mechanism of solid materials with the characteristics of ultrasonic wave propagation for numerical simulation has gradually gained attention. For example, Scalerandi et al. [46,47] conducted numerical simulations of ultrasonic propagation in locally damaged materials and provided theoretical explanations for acoustic resonance frequency drift and slow-motion phenomena using P–M spatial models. Shen and Cesnik [48,49] proposed a parallel algorithm based on the local interaction simulation method and penalty function method to simulate the “contact impact” behavior during the interaction between nonlinear ultrasonic Lamb waves and fatigue cracks. It was found that the nonlinear interaction of Lamb waves with fatigue cracks generates high harmonics and DC components. Yan et al. [50] used finite element numerical simulation to obtain transient maps of ultrasonic acoustic field propagation at different stages of crack extension in fatigue specimens of SLM 316L stainless steel. As shown in Figure 2, with the increase in crack length, the energy of diffracted waves gradually decreases, and the energy of the reflected waves from cracks gradually increases. Especially when the crack length reaches 230 μm, ultrasonic waves mainly diffract through the upper and lower tips of the crack. The energy of diffracted waves passing through the crack surface is already very weak, and the energy of ultrasonic waves reflected from the crack is relatively large. The nonlinear phenomenon of ultrasonic waves is mainly caused by the “breathing effect” generated by the diffraction of ultrasonic waves on the crack surface. When the crack length increases to 210 μm, the energy of the diffracted wave on the crack surface decreases, and the closed area generated by the “breathing effect” decreases accordingly. The nonlinear effect between the crack and ultrasonic wave becomes weaker, and the relative nonlinear coefficient decreases. As can be seen, the combination of numerical simulation and nonlinear ultrasound theory is an effective way to clarify the correlation between mesoscopic damage evolution and the nonlinear ultrasonic response of complex structured SLM metal parts.

### 2.3. Research on Processing Algorithms and Feature Extraction for Damage Assessment Applicable to Nonlinear Ultrasound Detection Signals

It is important to study the processing algorithms and damage evaluation feature extraction used for nonlinear ultrasonic detection signals due to the influence of temperature, shock, mechanical vibration, and other noise on the detection results during the detection process. The relevant methods are mainly divided into two categories: (1) Using different signal processing methods to eliminate noise interference and extract feature indicators for damage assessment. Collison et al. [51,52] performed a time-frequency analysis of ultrasonic detection signals using continuous wavelet transformation and selected the average power of the low-order side-frequency components as an indicator of damage; Yan et al. [53,54] proposed a new signal processing EMD-ESI-FFT method to extract nonlinear coefficients as damage indicators; and Masuda et al. [55] used the Hilbert transform to extract the amplitude modulation intensity as an indicator of damage. (2) Nonlinear ultrasound and time-reversal methods are combined to achieve sensitive identification and accurate localization of the damage. Time reversal refers to the calculation of the time reversal of the signal emitted from the sound source after it is received by the transducer array, i.e., the signal *s*(*t*) is changed to *s*(−*t*), and then re-emitted by the corresponding receiving transducer unit, respectively. Signals emitted from different arrays and propagated along different paths will arrive at the position of the sound source at the same time, resulting in a superposition of focusing. Wan [56] applied the time-reversal method to nonlinear coefficient extraction, and the experimental results showed that the energy of the second-harmonic signal doubled after pulse-reversal processing. Goursolle [57] used a P–M spatial model to simulate the time-reversal sound field in a solid plate. The results showed that when using the high-order harmonic time-reversal method, the time-reversal signal formed a focus at the nonlinear damage location, and damage localization could be achieved based on the distribution of time-reversal sound wave energy. Extracting appropriate feature indicators is crucial for evaluating the damage of SLM metal parts based on a nonlinear ultrasonic response. The feature indicators should not only accurately reflect the damage evolution process of the part but also exclude the influence of interference noise. Due to the complexity of the interaction mechanism between ultrasonic waves and mesoscopic damage in SLM metal parts, there is a lack of more practical methods for extracting feature indicators. Therefore, how to extract suitable damage indicators is a problem that requires in-depth research.

In summary, it is scientifically feasible to establish a failure mechanism model for SLM metal parts based on the ultrasonic nonlinear response to track the mesoscopic damage evolution of SLM metal parts and to clarify the cross-scale coupling mechanism between the mesoscopic damage evolution and the evolution of the macroscopic service state. However, due to the complexity of the interaction mechanism between ultrasonic waves and mesoscopic damage in SLM metal parts, as well as the complex service conditions faced by SLM metal parts, such as varying loads, working conditions, and strong impacts, it is difficult to match the constructed failure mechanism model with complex practical situations. The real-time collection and storage of “big data” by the component service safety monitoring system is a “sleeping treasure”. Therefore, fully utilizing the effective information hidden within the monitoring “big data” to further improve the mechanism model is an effective way to solve the problem.

## 3. Data-Driven Approach for Evaluating the Service Status of Metal Parts

With the rapid development of big data, artificial intelligence, and sensor technology, data-driven approaches for evaluating the service status of metal parts [58,59,60] have received much attention from scholars, and thus, related technologies are flourishing. The relevant research approaches can be summarized as follows

### 3.1. Machine Learning Approaches

The main idea of the machine learning approach is to fit the evolution law of performance degradation variables of metal parts through machine learning and predict the remaining life of metal parts (quantified degradation state of service performance) by rolling extrapolation to the failure threshold or to directly establish a mapping relationship between monitoring data and failure time to predict the remaining life of the metal part. Typical methods of machine learning include neural networks, support vector machines (RVM), Bayesian networks, random forests, extreme learning machines, etc. For example, Wang et al. [58] used a multi-scale attention convolutional network to predict the remaining service life of milling cutters. By constructing a self-attention model, they effectively fused the input multi-sensor monitoring data, designed a multi-scale learning strategy to automatically extract degradation indicators at different time scales, and combined it with a multi-layer perceptron to predict the remaining service life of milling cutters. Wu et al. [59] used the random forest method to predict tool wear life based on the experimental data collected from 315 milling experiments.

Machine learning-based prediction has significant advantages in the automatic extraction of deep indicators from big data, complex structural data fitting, nonlinear mapping, etc. However, the “implicit” mapping relationship between monitoring data and failure time, established by the machine learning network, is not clear in physical meaning, which can reflect the specific characteristics of the monitoring metal part but cannot explain the mechanism of the metal part’s degradation and failure. Although it can reflect the specific characteristics of the monitoring of metal parts, it cannot explain the degradation failure mechanism of the metal parts. In addition, the selection of hyper-parameters in machine learning models has an important impact on the accuracy and robustness of the prediction results, and how to reasonably select the hyper-parameters of machine learning approaches is a very challenging problem. Machine learning approaches can only obtain deterministic residual life prediction values, and there are deficiencies in the ability to quantify the prediction uncertainty, and the inability to quantify the prediction uncertainty also means the inability to quantify the risk of the prediction results.

### 3.2. Statistical Data-Driven Approaches

The statistical data-driven approach is based on probability and statistics theory and establishes a performance degradation variable evolution law model in a random model framework. It can not only obtain the point estimate of the remaining life but can also describe the uncertainty of the prediction (confidence intervals, variance, and other quantitative indicators of uncertainty), which is extremely important for the establishment of personalized and precise health management of parts and scientific decision-making. The relevant approaches can be divided into two categories: (1) Describe the evolution of individual performance degradation variables through explicit stochastic process models such as the Wiener process, Gamma process, Markov chain, hidden Markov process, etc. [60,61], and realize the estimation of the model parameters through the monitoring data, so as to quantitatively describe the uncertainty of the residual life prediction in the form of probability distribution. (2) Use the multidimensional variables that characterize the performance degradation of the metal part to establish an explicit model to describe the uncertainty of the remaining life prediction. (ii) Use the multidimensional variables characterizing the equipment’s performance to establish an explicit stochastic model so that the model can reflect the degradation process of the metal part as comprehensively as possible. There are two main ideas of multivariate coupling; one is based on the Copula function [62,63], which integrates the marginal distribution of each degradation variable and the Copula function into an overall distribution. The second is to transform the multidimensional data projection into one-dimensional data through optimization, weighting, fusion filtering, and other information fusion approaches [64,65,66].

The statistical data-driven method has a natural advantage in quantifying the uncertainty of remaining life prediction. The random model parameters are closely related to the degradation and failure process of metal parts, making the model interpretable. However, its ability to process monitoring big data, which has characteristics such as large differences in multi-source signals, multiple sampling strategy forms, and a low data value density, is very limited. In addition, based on a single performance degradation variable, it is not possible to comprehensively characterize the health status of parts under complex operating conditions. The existing basic idea of modeling multivariate stochastic processes is to convert multivariate variables into single variables, which does not fully consider the mechanism of multivariate coupling and mutual influence. Moreover, the problem of solving the remaining life distribution caused by multivariate coupling has not been effectively solved.

### 3.3. The Combination of Machine Learning and Statistical Data-Driven Approaches

Combining the machine learning approaches with statistical data-driven approaches is expected to integrate the advantages of both and compensate for their respective limitations. For example, Yu et al. [67] used transfer learning techniques to extract the performance degradation features, established a Gaussian process regression prediction model, and finally updated the parameters of the Gamma degradation model based on the predicted values, achieving a reliability evaluation and remaining life prediction of a part. Duan et al. [68] used an asymmetric dual channel autoencoder to extract the performance degradation features, constructed health indicators in an unsupervised manner, and introduced a nonlinear Wiener process (NWP) to construct a device degradation evolution model. The NWP was used to calculate the remaining life probability density function, and the expected value on the confidence interval was calculated to obtain a remaining life prediction containing uncertainty estimates. However, it cannot be ignored that such methods have some limitations. Firstly, the degraded features extracted by machine learning and the modeling of stochastic processes are only a simple combination relationship, which cannot guarantee that the extracted degraded features can adapt to and match the stochastic process model used. Secondly, the challenge is that the degradation indicators extracted from monitoring big data through machine learning do not have clear physical meanings, and how to reasonably determine the failure threshold based on the degradation indicators has become a new problem.

**Figure 3:**
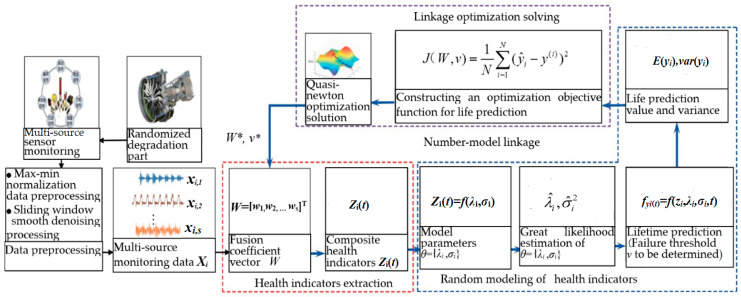
The number-model linkage flowchart for predicting the remaining life of the randomly degraded metal parts.

How can the degraded indicators extracted by machine learning adapt and match the random process model used? Some scholars have conducted exploratory research. Zhang et al. [69] proposed a solution for predicting the remaining life of randomly degraded metal parts through a number-model linkage solution. “Number” refers to the extraction of degraded features from data, while “model” refers to the random modeling of the time-varying evolution process of the extracted degraded indicators. Figure 3 shows the number-model linkage flowchart for predicting the remaining life of randomly degraded metal parts. Based on the multi-source sensor monitoring data of the equipment, a composite health index is constructed by the weighted fusion of multiple sensors at the data layer to characterize the degradation characteristics of the equipment. Then, a stochastic process model is used to characterize the time-varying evolution trend of the composite health index. The life value prediction is achieved by solving the time when the first failure threshold of the composite health index is reached. Based on the deviation between the predicted life value and the actual life of the equipment, an optimization objective function is constructed to characterize the prediction effect. The fusion coefficient of multiple sensors and the parameters in the stochastic degradation modeling are inversely optimized and adjusted to form a feedback loop between the extraction of the composite health index and the stochastic degradation modeling, achieving the goal of automatic matching between the composite health index and the stochastic model. The experimental results show that compared with typical machine learning approaches and combination approaches, such as support vector machines, convolutional neural networks, deep belief networks, improved particle filtering algorithms, recurrent neural networks, autoencoders, etc., the remaining life prediction reliability of the number-model linkage approach is higher. However, the drift coefficient estimation results of this approach ignore the data of the intermediate evolution process, which will inevitably affect the predictive performance of the model. Secondly, when optimizing the objective function, it only considers the point estimation of the remaining life prediction without considering the variance of the remaining life prediction that reflects prediction uncertainty. Therefore, further in-depth research is needed to provide more optimized solutions.

### 3.4. Mechanism Model Combined with Data-Driven Approach

The main method of mechanism modeling is to construct parameterized mathematical models that describe the degradation process of parts based on failure mechanisms. Existing mechanism modeling approaches make it difficult to accurately reflect the actual service status of parts under nonlinear and complex conditions. The feasible solution to this problem is to fully utilize the real-time operational monitoring of big data of the specific part and combine the mechanism model with data-driven approaches. This type of method can be divided into two categories: (1) constructing a degradation state measurement model based on mechanism models that are based on monitoring big data, using Kalman filtering, particle filtering, and other methods to update the degradation state and mechanism model parameters, and thus achieving a part’s remaining life prediction [70,71,72]. (2) Based on the data-driven and mechanistic models, predict the remaining life of the part separately and integrate the remaining life prediction results of the two models using decision layer fusion approaches [73,74,75,76]. Although the combination of mechanism models and data-driven approaches has made some progress, if the combination of the two types of models is not reasonable, it will introduce new errors and thus, cannot improve the reliability of the remaining life prediction results of metal parts. Secondly, due to the nonlinear and complex characteristics of a metal part’s operating conditions in practical applications, it is difficult to model an accurate mechanism model that characterizes the evolution law of its service state, or the cost of obtaining a mechanism model is too high, which, to some extent, limits the promotion and application of such approaches.

## 4. Problem Analysis and Solution Exploration

From the current research status, the personalized and accurate health management of service metal parts is a frontier research direction driven by engineering demands, and there are few reports on an intelligent assessment of the service state of SLM metal parts under nonlinear and complex operation conditions. The existing theories and methods still have many deficiencies in solving residual life predictions and achieving intelligent assessments of the service state of service parts, and the related problems need to be studied in depth and systematically.

**Figure 4:**
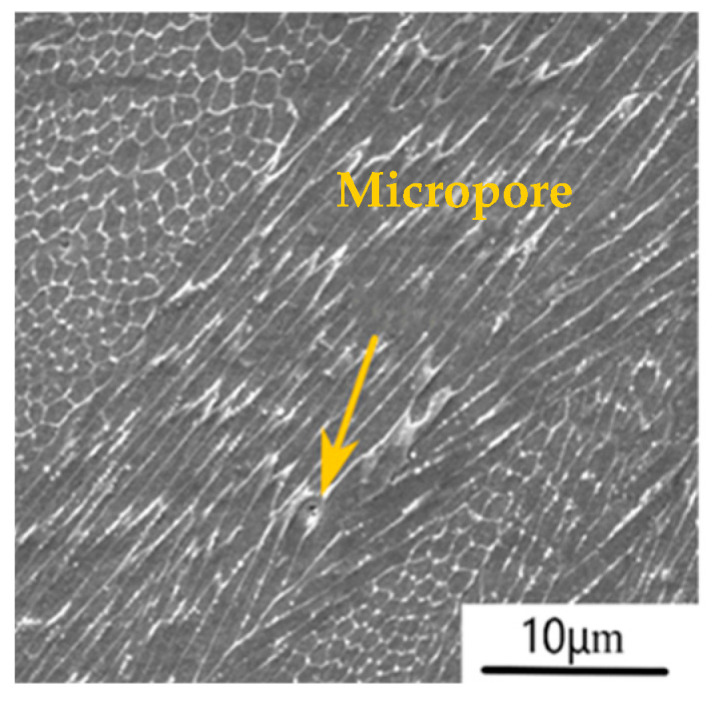
Micropore.

**Figure 5:**
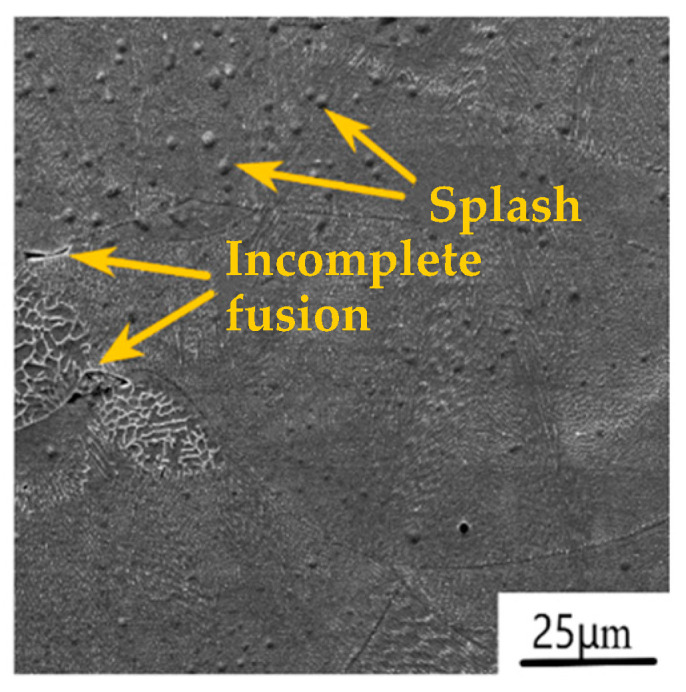
Splash and incomplete fusion.

SLM additive manufacturing technology can form functional parts with almost 100% density of any complex structure. It has a very wide range of application prospects in fields such as aerospace, medical equipment, and the automotive industry. However, the process of forming SLM metal parts is a complex metallurgical process. Due to the absorption of high-energy laser beam energy by the metal powder, the temperature rapidly rises, causing the metal powder to melt and solidify quickly. The forming process is also affected by factors such as material properties, process parameters, and equipment conditions. Even if the strength requirements are met, initial micro defects are inevitably present inside SLM metal parts [77,78,79,80]. Figure 4 and Figure 5 show the micropore, splash, and incomplete fusion defects during SLM 316L stainless steel forming. When the energy of the laser beam is too low, the melting time of the metal powder is too long, and it does not solidify quickly. The gas carried in the metal powder does not overflow, forming micro-hole defects. When the laser scanning speed is low, and the instantaneous energy of the high-energy laser beam is too high, the temperature in the fusion zone rapidly increases, and the metal powder quickly melts to form liquid metal. The liquid metal partially vaporizes, causing gas-phase expansion between powders and back impact force. Under the action of the back impact force, the metal–liquid phase is impacted and splashes, forming splashing defects. Therefore, under nonlinear and complex operating conditions, these micro defects will inevitably evolve gradually and macroscopically manifest as the gradual degradation of the service performance and evolution of the service state of SLM metal components. Therefore, tracking the mesoscopic damage evolution status of SLM metal parts, accurately evaluating their service status (health status, quantified performance degradation status (remaining life estimation), and failure status), and implementing personalized and precise health management (normal maintenance, remanufacturing repair, reserved parts, and scrap) at the “precise timing” of the evolution of the part’s service status can reduce costly unplanned maintenance and achieve the goal of saving manufacturing costs and providing environmental benefits at the same time.

As can be seen from the above analysis, the classical mechanism modeling method has the advantage of highly summarizing the universal laws of equipment failure. It is the crystallization of human wisdom in basic disciplines, such as mechanics, physics, chemistry, and materials, and has a solid scientific foundation. Therefore, the research team believes that while revealing the scientific principles of the universality of part failure, by using “measurement big data” to further fully express the “personalized” implicit characteristics of metal parts, it is expected that a construct of a “chimeric” model with scientific universality and high compatibility with complex practical situations will improve the reliability of the service status assessment of SLM metal parts in service. However, there are still some challenges when specifically applying this method to solve the problem of service state evolution and the life prediction of SLM metal parts under nonlinear and complexity conditions. These challenges can be summarized as follows: (1) The evolution of the service state of SLM metal parts is closely related to the mesoscale damage evolution. How does the “chimeric” model reflect the coupling mechanism between mesoscale damage evolution and macroscopic service state evolution? (2) How do we organically integrate the failure mechanism model with the “personalized” information of the parts hidden in monitoring big data when constructing a “chimeric” model? (3) How do we avoid the blindness of a model’s hyperparameter selection and nonlinear optimization problems, such as local optimization, when mining the effective information implied by monitoring big data? (4) How do we achieve an online optimization evaluation of the service status based on offline training of “chimeric” models? Therefore, establishing a scientifically feasible “chimeric” model is an important means to ensure the safety of SLM metal parts in practical applications. In terms of realizing the hybrid driving of the mechanism model and data, Wang et al. [81] used a correlation vector machine with different kernel parameters to regress the rolling bearing health indexes, then fitted the correlation vectors using an exponential mechanism model, and dynamically evaluated the fitted degradation curve by calculating the Frechette distance between the fitted degradation curve and the smoothed sequence of health indicators to obtain the optimal degradation curve and its corresponding degradation model parameters. Then, they recursively pushed the health indicators until they reached the failure threshold to achieve the prediction of the remaining life of the rolling bearing. Xie et al. [82] published a review paper in the *Journal of Mechanical Engineering* in 2022, discussing the problem of “fusion optimization in the field of lithium battery residual life prediction” and pointing out that the integration of mechanistic models into data-driven battery life prediction algorithms can improve the interpretability of the life prediction results, but their computational complexity is usually higher. However, the computational complexity is usually high. How to propose a more reasonable fusion framework to improve the computational efficiency of the algorithm is a key issue to be solved in the future. However, these studies currently cannot be directly applied to the intelligent evaluation method and theoretical framework for the service status of SLM metal parts based on the “chimeric” model.

**Figure 6:**
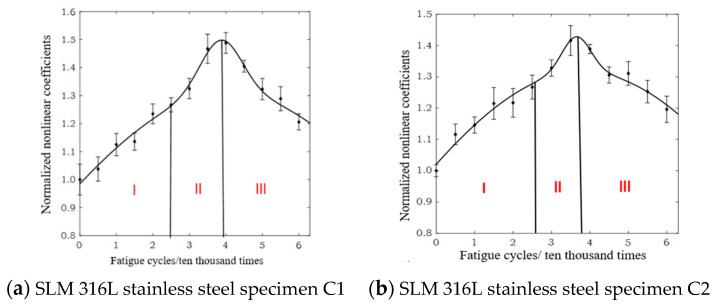
Three stages of the normalized ultrasonic nonlinear coefficient–fatigue cycles curve [98].

**Figure 7:**
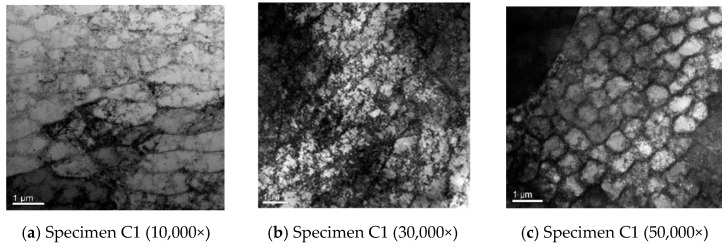
The TEM photographs of the SLM 316L stainless steel specimen C1 were subjected to different fatigue cycles [95].

Yan et al. [50,53,54] have conducted extensive research on the failure mechanism of additively manufactured metal parts, numerical simulation and experimental study of ultrasonic inspection, ultrasonic inspection signal processing for nonlinear complex systems, and data-driven remaining life prediction. The research [50,53,54] indicates that the macroscopic damage and failure behavior of SLM metal parts originate from the deformation and damage evolution at the mesoscale inside the parts. The use of an ultrasonic nonlinear response-driven mesoscopic damage evolution tracking method to clarify the cross-scale coupling mechanism between mesoscopic damage evolution and the macroscopic service state evolution of SLM metal parts is scientifically feasible [83,84,85,86,87,88,89,90,91]. Due to the fact that nonlinear ultrasonic testing can essentially reflect the influence of tiny defects (micrometer level) inside metal materials using the ultrasonic propagation process [92,93,94], and the numerical simulation technology of nonlinear ultrasonic testing is relatively mature, Yan et al. [53,54] used numerical simulation methods to deeply study the physical mechanism of the nonlinear response generated by the interaction between ultrasonic waves and the mesoscopic damage of SLM 316L stainless steel parts. Combined with high-cycle fatigue loading experiments and nonlinear ultrasonic testing experiments, they systematically explored the method for accurately describing and characterizing the service state evolution of SLM 316L stainless steel parts based on ultrasonic nonlinear responses, established a failure mechanism model for the tested specimen, and used the established model to predict the remaining life of SLM 316L stainless steel specimens. Not only does it solve the problem of high costs in obtaining failure mechanism models, but also, the ultrasonic nonlinear response characteristic parameters also have clear physical meanings, and the interpretability of the established model is strong. Figure 6 and Figure 7 [95] show the partial research results. The normalized ultrasonic nonlinear coefficient is sensitive to the fatigue damage degree of SLM 316L stainless steel parts that is caused by fatigue loading. The relationship between the normalized ultrasonic nonlinear coefficients and the fatigue cycles was roughly in a mountain-shaped curve. The variations in the mountain-shaped curve can be divided into three stages. In the first stage, the multiplication of planar dislocation is the reason for the increase in the normalized ultrasonic nonlinear coefficients. In the second stage, with the accumulation of the fatigue degree, a microcrack appears and gradually propagates, and the thicker dislocation vein gradually evolves into a more dense dislocation wall so that the normalized ultrasonic nonlinear coefficients accelerate and reach the maximum value. In the third stage, due to the propagation of the microcrack into a macrocrack, the normalized ultrasonic nonlinear coefficients gradually decrease. To enhance the generalization ability of the model, it is necessary to organically integrate the “personalized” information of the parts hidden in monitoring big data into the failure mechanism model, construct a “chimeric” model, and quantitatively evaluate and optimize the model prediction results online in practical applications. The posterior distribution of the latent variables in a given variational family for the probability model can be iteratively estimated by the variational Bayesian method [96,97,98,99,100,101]. Therefore, based on the powerful data information description ability of machine learning and the scientific foundation of mechanism modeling, starting from the basic principles of Bayesian estimation, it is expected to fundamentally solve the problem of uncontrollable service status and unsafe service of SLM metal parts under nonlinear and complex conditions.

**Figure 8:**
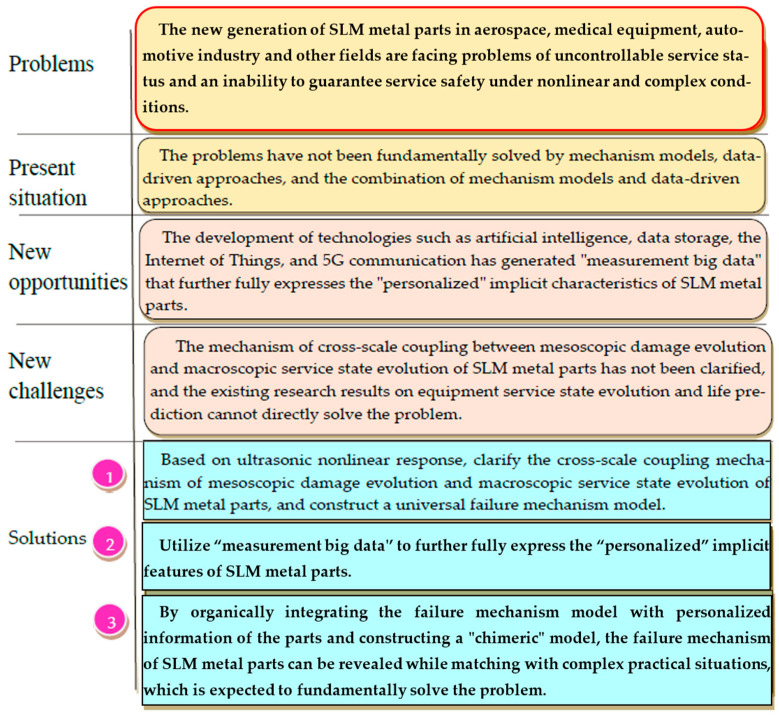
The research outline.

The evolution mechanism of the service life and service safety of SLM metal parts come from practical applications, and corresponding theoretical research must also serve the application. Based on the nonlinear response of the ultrasound, constructing a cross-scale correlation model between the mesoscopic damage evolution and macroscopic service state evolution of SLM metal parts is helpful for a deeper understanding of the mesoscopic damage dynamics’ evolution that is driven by ultrasound nonlinear responses and revealing the failure mechanism of SLM metal parts. In today’s era of fully developed artificial intelligence, such as big data and 5G communication, there are sufficient basic conditions for constructing a theoretical framework to solve this problem and researching effective solutions. Measuring “big data” resources can provide a new avenue for research on the evolution mechanism of service life and service safety of SLM metal parts, but it still requires “exploring new paths and forging ahead”. Related research has promoted the cross-fusion of metal physics, nonlinear ultrasound, and artificial intelligence. By organically integrating the failure mechanism model of SLM metal parts with the “personalized” information hidden in monitoring big data, a “chimeric” model suitable for intelligent evaluation of the service status of SLM metal parts is studied, fundamentally solving the problem of uncontrollable service status and the inability to guarantee the service safety of SLM metal parts under nonlinear and complex conditions (Figure 8 shows the research outline). The relevant research results have great theoretical research value and practical application significance, which will promote the deep integration of big data artificial intelligence methods with intelligent manufacturing, aerospace, medical equipment, the automotive industry, and other industries. It is a key supporting technology for strategic emerging industries that needs to be fully developed.

## 5. Summary and Prospect

Due to the nonlinear, strongly coupled, and time-varying characteristics of the SLM parts’ manufacturing process, the micro defects retained during the laser powder fusion process determine that SLM metal parts will inevitably undergo mesoscopic damage evolution and macroscopic service state evolution during service. Therefore, the primary issue in improving the service safety of SLM components is to clarify the cross-scale coupling mechanism between the mesoscopic damage evolution and macroscopic service state evolution. Nonlinear ultrasonic testing has the advantages of being non-destructive, fast, and with a wide detection range, which can fundamentally reflect the influence of small defects inside materials on the ultrasonic propagation process. In recent years, scholars at home and abroad have achieved many valuable research results based on nonlinear ultrasonic evaluations of metal material damage (fatigue, aging, creep, etc.). The mesoscopic damage evolution process of SLM metal parts is tracked based on the ultrasonic nonlinear response in the proposed approach, and the cross-scale coupling mechanism between mesoscopic damage evolution and macroscopic service state evolution will be clarified. On this basis, a “chimeric” model will be constructed that organically integrates the failure mechanism with the “personalized” information of SLM parts. The “chimeric” model utilizes the powerful data processing capabilities of deep learning networks to condense universal scientific laws of the evolution of the service status of parts from ultrasonic monitoring data. At the same time, it further fully expresses the “personalized” hidden characteristics of the SLM metal parts through the on-site monitoring of “big data”, which can inherit the scientific framework of existing classical theories and explore the effective information hidden in monitoring “big data”, thereby finding new breakthroughs to solve problems. The proposed approach and technical roadmap in this paper are scientific and operable and can effectively solve problems of uncontrollable service states and the inability to ensure the service safety of SLM metal parts under nonlinear and complex operating conditions. Future research work mainly includes:

(1) The mesoscopic damage evolution process of SLM metal parts is tracked based on an ultrasonic nonlinear response, and the cross-scale coupling mechanism between mesoscopic damage evolution and macroscopic service state evolution will be clarified. Build a failure mechanism model based on cyclic Bayesian deep learning to achieve the clustering-inversion process of ultrasonic monitoring data, service status evaluation, and ultrasonic monitoring data estimation and construct a regularized loss function based on the difference between monitoring data and its estimated value and the hyperparameter σ to ensure the feasibility of intelligent evaluation of the SLM metal parts’ service status based on ultrasonic monitoring data.
(2) Construct a “chimeric” model that organically integrates failure mechanism and personalized information of the SLM parts, design the mechanism for the organic fusion of failure mechanism model and personalized information of the SLM parts, optimize the loss function during the training process of the “chimeric” model using system noise characteristic information, enhance the interpretability and generalization ability of the model, and enable the “chimeric” model to match complex practical situations while revealing the scientific mechanism of SLM metal part failure.
(3) The optimization process based on the Bayesian recursive estimation will be used to construct the operational mechanism of the “service state evaluation network”, which realizes the intelligent prediction and updates the process of the service state of the SLM metal parts based on the “chimeric” model. The powerful nonlinear fitting ability of deep learning will be combined with the Bayesian recursive estimation optimization process to achieve complementary advantages and ensure that the “chimeric” model converges to an effective solution, realizing the intelligent evaluation of the service state of SLM metal parts under nonlinear and complex operating conditions.
(4) Based on Bayesian estimation theory, combined with the offline training of the “chimeric” model operation mechanism and Bayesian recursive optimization strategy, quantitative evaluation indicators for the service status assessment effect of the “chimeric” model will be derived. This can theoretically reveal that the intelligent service status assessment approach, based on the “chimeric” model, can fundamentally solve the essential reasons for the service safety problems of SLM metal parts under nonlinear and complex conditions and enhance the generalization ability of the “chimeric” model.

## Data Availability

The data presented in this study are available upon request from the corresponding author.

## References

[1] Yan X.L., Tang X.J. (2023). A novel method for early fatigue damage diagnosis in 316L stainless steel formed by selective laser melting technology. Materials.

[2] Liu B., Zhao Z.Y., Bai P.K., Liang M.J., Guan R.G. (2019). The compressive behavior of a porous 316L stainless steel prepared by selective laser melting. Lasers Eng..

[3] Kuznetsov P.A., Shakirov I.V., Bobyr’ V.V., Zhukov A.S., Klimov V.N. (2020). Features of Melt Gas Atomization and Selective Laser. Melting of High-Strength Austenitic Nitrogen-Containing Steel Powders. Met. Sci. Heat Treat..

[4] The Cause of the 16 Death Accident of a US Transport Plane Has Been Determined: The Propeller Suddenly Flew Out and Cut the Fuselage in Half. https://baijiahao.baidu.com/s?id=1619294551541955092&wfr=spider&for=pc.

[5] Le K., Wong C., Chua K., Tang C., Du H. (2020). Discontinuity of overhanging melt track in selective laser melting process. Int. J. Heat Mass. Transf..

[6] Qiu C.L., Kindi M.A., Aladawi A.S., Hatmi I.A. (2018). A comprehensive study on microstructure and tensile behavior of a selectively laser melted stainless steel. Sci. Rep..

[7] Boschetto A., Bottini L., Ghanadi N. (2022). Areal analysis investigation of selective laser melting parts. J. Manuf. Mater. Process..

[8] Nandhakumar R., Venkatesan K. (2023). A process parameters review on selective laser melting-based additive manufacturing of single and multi-material: Microstructure, physical properties, tribological, and surface roughness. Mater. Today Commun..

[9] Lv J.L., Zhou Z.P., Wang Z.Q. (2022). Comparing the sensitization behavior and the corrosion resistance of the wrought and selective laser melted 316L stainless steels. Mater. Lett..

[10] Wu S.L., Wang S.J., Wang H.T. (2024). Research progress in selective laser melting of refractory metals. Rare Met. Mater. Eng..

[11] Erutin D.P., Popovich A.A., Sufiiarov V.S. (2024). Effect of scanning angle on density of 1cp/copper selective laser melted composite. Inorg. Mater.-Appl. Res..

[12] Wang Y.F., Yu J., Wang Z.S. (2023). Surface quality improvement at selective laser melting AlSi10Mg by optimizing single point diamond turning parameters. Mater. Test..

[13] Yan X.L., Dong S.Y., Xu B.S., Cao Y. (2018). Progress and Challenges of Ultrasonic Testing for Stress in Remanufacturing Laser Cladding Coating. Materials.

[14] Yan X.L., Xu X.S. (2021). Method for accurately measuring of acoustic time difference based on optimal threshold. Measurement.

[15] Janousek L., Stubendekova A., Smetana M. (2018). Novel insight into swept frequency eddy-current non-destructive evaluation of material defects. Measurement.

[16] Rusli N.S., Abidin I.Z., Aziz S.A. (2016). A review on eddy current thermography technique for non-destructive testing application. J. Teknol..

[17] Plessis A.D., Roux S.G., Guelpa A. (2016). Comparison of medical and industrial X-ray computed tomography for non-destructive testing. Case Stud. Nondestruct. Test. Eval..

[18] Kolkoori S., Wrobel N., Zscherpel U., Ewert U. (2015). A new X-ray backscatter imaging technique for non-destructive testing of aerospace materials. NDT E Int..

[19] Kutman M.K., Muftuler F.Z.B., Harmansah C., Guldu O.K. (2020). Use of bacteria as fluorescent penetrant for Penetrant Testing (PT). J. Nondestruct. Eval..

[20] Shelikhov G.S., Glazkov Y.A. (2011). On the improvement of examination questions during the nondestructive testing of magnetic powder. Russ. J. Nondestruct. Test..

[21] Yan H.J., Xu C.G., Xiao D.G., Cai H.C. (2016). Research on nonlinear ultrasonic properties of tension stress in metal materials. J. Mech. Eng..

[22] Jang J., Sohn H., Lim H.J. (2023). Spectral noise and data reduction using a long short-term memory network for nonlinear ultrasonic modulation-based fatigue crack detection. Ultrasonics.

[23] Solodov I.Y. (1998). Ultrasonics of non-linear contacts: Propagation, reflection and NDE-applications. Ultrasonics.

[24] Nazarov V.E., Sutin A.M. (1997). Nonlinear elastic constants of solids with cracks. Acoust. Soc. Am. J..

[25] Jasiūnienė E., Mažeika L., Samaitis V., Cicėnas V., Mattsson D. (2019). Ultrasonic non-destructive testing of complex titanium/carbon fibre composite joints. Ultrasonics.

[26] Zhou Z.G., Liu S.M. (2011). Nonlinear ultrasonic Techniques Used in Nondestructive Testing: A Review. J. Mech. Eng..

[27] Liu S.J., Lee M., Choi C., Shin K. (2023). Effect of additive manufacturing of SUS316L using selective laser melting. J. Mater. Res. Technol.-JMYT.

[28] Zhou Y., Zhang K., Liang Y.R., Cheng J., Dai Y.L. (2022). Selective laser melted magnesium alloys: Fabrication, microstructure and property. Materials.

[29] Rusin N.M., Skorentsev A.L., Akimov K.O. (2023). Al-40Sn alloy produced by selective laser melting of elemental powder mixtures. Phys. Met. Metallogr..

[30] Wang Q.Y., Gao M.D., Li Q., Liu C.H., Li L., Li X.Y., Liu Z.F. (2024). A Review on energy consumption and efficiency of selective laser melting considering support: Advances and prospects. Int. J. Precis. Eng. Manuf.-Green Technol..

[31] Walker K.F., Liu Q., Brandt M. (2017). Evaluation of fatigue crack propagation behavior in Ti-6Al-4V manufactured by selective laser melting. Int. J. Fatigue.

[32] Bao H.Y.X., Wu S.C., Wu Z.K., Kang G.Z., Peng X., Withers P.J. (2021). A machine-learning fatigue life prediction approach of additively manufactured metals. Eng. Fract. Mech..

[33] Chen Y.F., Wang X.W., Shen J.W., Peng Y.W., Jiang Y., Yang X.Y., Leen S.B., Gong J.M. (2022). Deformation mechanisms of selective laser melted 316L austenitic stainless steel in high temperature low cycle fatigue. Mater. Sci. Eng. A-Struct. Mater. Prop. Microstruct. Process..

[34] Shi Q., Hu C.H., Si X.S., Hu X.X., Zhang Z.X. (2019). Remaining useful lifetime prediction method of controlled systems considering performance degradation of actuator. Acta Autom. Sin..

[35] Liao L.X., Köttig F. (2014). Review of hybrid prognostics approaches for remaining useful life prediction of engineered systems, and an application to battery life prediction. IEEE Trans. Reliab..

[36] Cantrell J.H., Zhang X.G. (1998). Nonlinear acoustic response from precipitate-matrix misfit in a dislocation network. J. Appl. Phys..

[37] Suzuki T., Hikata A., Elbaum C. (1964). An harmonicity due to glide motion of dislocations. J. Appl. Phys..

[38] Cantrell J.H. (2009). Ultrasonic harmonic generation from fatigue-induced dislocation substructures in planar slip metals and assessment of remaining fatigue life. J. Appl. Phys..

[39] Cantrell J.H., Yost W.T. (1994). Acoustic harmonic generation from fatigue-induced dislocation dipoles. Philos. Mag..

[40] Breazeale M.A., Thompson D.O. (1963). Finite-amplitude ultrasonic waves in aluminum. Appl. Phys. Lett..

[41] Granato A., Lucke K. (1956). Theory of mechanical damping due to dislocations. J. Appl. Phys..

[42] Cantrell J.H., Yost W.T. (2001). Nonlinear ultrasonic characterization of fatigue microstructures. Int. J. Fatigue.

[43] Pecorari C. (2003). Adhesion and nonlinear scattering by rough surfaces in contact: Beyond the phenomenology of the Preisach-Mayergoyz framework. J. Acoust. Soc. Am..

[44] Baltazar A., Rokhlin S.I., Pecorari C. (2002). On the relation between ultrasonic micro mechanic properties of contacting rough surfaces. J. Mech. Phys. Solids.

[45] McCall K.R., Guyer R.A. (1994). Equation of state and wave propagation in hysteretic nonlinear elastic materials. J. Geophys. Res..

[46] Delsanto P.P., Scalerandi M. (2003). Modeling nonclassical nonlinearity, conditioning, and slow dynamics effects in mesoscopic elastic materials. Phys. Rev. B.

[47] Gliozzi A.S., Nobili M., Scalerandi M. (2006). Modeling localized nonlinear damage and analysis of its influence on resonance frequencies. J. Phys. D Appl. Phys..

[48] Shen Y., Cesnik C.E.S. (2017). Modeling of nonlinear interactions between guided waves and fatigue cracks using local interaction simulation approach. Ultrasonics.

[49] Shen Y., Cesnik C.E.S. (2019). Nonlinear scattering and mode conversion of Lamb waves at breathing cracks: An efficient numerical approach. Ultrasonics.

[50] Yan X.L., Dong S.Y., Xue N., Wang W.L. (2016). Numerical simulation of ultrasonic propagation and defect testing in laser cladding remanufacturing parts. Chin. Sci. Bull..

[51] Collison I.J., Stratoudaki T., Clark M., Somekh M.G. (2008). Measurement of elastic nonlinearity using remote laser ultrasonics and cheap optical transducers and dual frequency surface acoustic waves. Ultrasonics.

[52] Vetrone J., Obregon J.E., Indacochea E.J., Ozevin D. (2021). The characterization of deformation stage of metals using acoustic emission combined with nonlinear ultrasonics. Measurement.

[53] Yan X.L., Xu X.S., Pan Q.X. (2020). Study on the measurement of stress in the surface of selective laser melting forming parts based on the critical refraction longitudinal wave. Coatings.

[54] Yan X.L., Wang H.P., Fan X.Z. (2023). Research progress in nonlinear ultrasonic testing for early damage in metal materials. Materials.

[55] Masuda A., Shinagawa T., Maekawa S., Kinugawa Y., Iba D., Sone A. (2007). Monitoring and evaluation of cracked beams based on nonlinear wave modulation. Sensor Systems and Networks: Phenomena, Technology, and Applications for NDE and Health Monitoring 2007.

[56] Wan C.H., Gang T., Liu B. (2015). Nonlinear ultrasonic evaluation of fatigue life of aluminum alloy welded joint based on pulse-inversion technique. Trans. China Weld. Inst..

[57] Goursolle T., Santos S.D., Matar O.B., Calle S. (2008). Non-linear based time reversal acoustic applied to crack detection: Simulations and experiments. Int. J. Non-Linear Mech..

[58] Wang B., Lei Y.G., Li N.P., Wang W.T. (2021). Multiscale convolutional attention network for predicting remaining useful life of machinery. IEEE Trans. Ind. Electron..

[59] Wu D.Z., Jennings C., Terpenny J., Gao R.X., Kumara S. (2017). A comparative study on machine learning algorithms for smart manufacturing: Tool wear prediction using random forests. J. Manuf. Sci. Eng..

[60] Zhang J.X., Hu C.H., He X., Si X.S., Liu Y., Zhou D.H. (2019). A novel lifetime estimation method for two-phase degrading systems. IEEE Trans. Reliab..

[61] Li T.M., Pei H., Pang Z.N., Si X.S., Zheng J.F. (2019). A sequential bayesian updated wiener process model for remaining useful life prediction. IEEE Access.

[62] Li J., Dai H.D., Jing B., Jiao X.X. (2021). A new dynamic-copula based correlated degradation feature for remaining useful life prediction. Chin. J. Electron..

[63] Jiang N., Liu Y., Deng Y., Yu F. (2020). Reliability assessment of concrete under chloride penetration and fatigue loading based on copula function. J. Mater. Civ. Eng..

[64] Kim M., Song C.Y., Liu K.B. (2019). A generic health index approach for multisensor degradation modeling and sensor selection. IEEE Trans. Autom. Sci. Eng..

[65] Liang W.G., Li C., Zhao L., Yan X.J., Sun S.Y. (2023). Summarization of remaining life prediction methods for special power plants. Appl. Sci..

[66] Lin J.D., Lin Z., Liao G.B., Yin H.P. (2021). A novel product remaining useful life prediction approach considering fault effects. IEEE-CAA J. Autom. Sin..

[67] Yu J., Oh H. (2023). AI-based degradation index from the microstructure image and life prediction models based on bayesian inference. Sustainability.

[68] Duan Y.H., Liu Z., Li H.H., Zhang C., Zhang N. (2023). A hybrid-driven remaining useful life prediction method combining asymmetric dual-channel autoencoder and nonlinear Wiener process. Appl. Intell..

[69] Zhang J.X., Zhang J.L., Zhang Z.X., Li T.M., Si X.S. (2024). Remaining useful life prediction for stochastic degrading devices incorporating quantization. Reliab. Eng. Syst. Saf..

[70] Chang Y.H., Hsieh Y.C., Chai Y.H., Lin H.W. (2023). Remaining-useful-life prediction for li-ion batteries. Energies.

[71] Yi Z.X., Zhao K., Sun J.R., Wang L.C., Wang K., Ma Y.Z. (2022). Prediction of the remaining useful life of supercapacitors. Math. Probl. Eng..

[72] Boukra T., Lebaroud A. (2019). New trend in enhancing bearing remaining useful life prediction. J. New Technol. Mater..

[73] Xia T.B., Shu J.Q., Xu Y.H., Zheng Y., Wang D. (2022). Multiscale similarity ensemble framework for remaining useful life prediction. Measurement.

[74] Zang Y., Wei S.G., Cai B.G., Wang H.S., Pecht M.G. (2021). Hybrid remaining useful life prediction method. A case study on railway D-cables. Reliab. Eng. Syst. Saf..

[75] Zhu K., Zhao X.W., Zhang L.M., Yu H. (2022). A hybrid method to predict the remaining useful life of scroll wheel of control rod drive mechanism. Sci. Technol. Nucl. Install..

[76] Cui Z.B., Jing B., Jiao X.X., Huang Y.F., Wang S.L. (2023). The integrated-servo-actuator degradation prognosis based on the physical model combined with data-driven approach. IEEE Sens. J..

[77] Shao K., Zhou Q.L., Liu Y., Wang C.F. (2022). Effect of laser power on the mechanical properties of selective laser melted 316L stainless steel. Laser Eng..

[78] Jeyaprakash N., Yang C.H., Prabu G., Radhika N. (2023). Mechanism correlating microstructure and wear behavior of Ti-6Al-4V plate produced using selective laser melting. Metal.

[79] Ghadhban A.H., Hasan I.H. (2022). Hardness and surface roughness of cobalt-chromium alloy produced by selective laser melting and casting techniques (An in vitro study). J. Res. Med. Dent. Sci..

[80] Hu Y., Chu C., Hu Y.Q., Zhang H.Y., Wang L.H., Zhang D. (2023). Numerical simulation and experimental research on the temperature field of selective laser melting of IN738LC alloy. Rare Met. Mater. Eng..

[81] Wang B., Lei Y.G., Li N.P., Li N.B. (2020). A hybrid prognostics approach for estimating remaining useful life of rolling element bearings. IEEE Trans. Reliab..

[82] Xu L., Deng Z.W., Xie Y., Hu X.S. (2022). Review on research progress in high-fidelity modeling, parameter identification and lifetime prognostics of lithium-ion battery. J. Mech. Eng..

[83] Jiao J.P., Lv H.T., He C.F., Wu B. (2017). Fatigue crack evaluation using the non-collinear wave mixing technique. Smart Mater. Struct..

[84] Liu P.P., Jang J.H., Sohn H. (2020). Crack localization by laser-induced narrowband ultrasound and nonlinear ultrasonic modulation. Smart Struct. Syst..

[85] Jang J., Liu P., Kim B., Kim S.-W., Sohn H. (2020). Silicon wafer crack detection using nonlinear ultrasonic modulation induced by high repetition rate pulse laser. Opt. Lasers Eng..

[86] Cao M.S., Lu Q.T., Su Z.Q., Radzienski M., Xu W., Ostachowicz W. (2022). A nonlinearity-sensitive approach for detection of “breathing” cracks relying on energy modulation effect. J. Sound Vib..

[87] Cao M.S., Su Z.Q., Deng T.F., Xu W. (2021). Nonlinear pseudo-force in “breathing” delamination to generate harmonics: A mechanism and application study. Int. J. Mech. Sci..

[88] Zhang F.Y., Li C., Tie Y., Yin Z.H. (2019). Experimental study on fatigue damage detection based on nonlinear ultrasonic. Fiber Reinf. Plast./Compos..

[89] Sufiyarov V.S., Borisov E.V. (2019). Effect of heat treatment modes on the structure and properties of alloy VT6 after selective laser melting. Met. Sci. Heat Treat..

[90] Shan S.B., Hasanian M., Cho H., Lissenden C.J., Cheng L. (2019). New nonlinear ultrasonic method for material characterization: Codirectional shear horizontal guided wave mixing in plate. Ultrasonics.

[91] Wang J.Z., Shen Y.F., Rao D.Y., Xu W. (2021). An instantaneous-baseline multi-indicial nonlinear ultrasonic resonance spectral correlation technique for fatigue crack detection and quantification. Nonlinear Dyn..

[92] Li W.B., Shi T.Z., Qin X.X., Deng M.X. (2021). Detection and location of surface damage using third-order combined harmonic waves generated by non-collinear ultrasonic waves mixing. Sensors.

[93] Zhang Q., Shen F., Fan F., Wang R., Wang Y., Niu H.J. (2020). A method for identifying false positive frequencies extracted from resonant ultrasound spectra for highly dissipative materials. J. Appl. Phys..

[94] Mora P., Chekroun M., Raetz S., Tournat V. (2021). Nonlinear generation of a zero group velocity mode in an elastic plate by non-collinear mixing. Ultrasonics.

[95] Qiao R., Yan X.L. (2022). The characterization of fatigue damage of 316L stainless steel parts formed by selective laser melting with harmonic generation technique. Materials.

[96] Ait-El-Fquih B., Hoteit I. (2022). Parallel- and cyclic-iterative variational bayes for fast kalman filtering in large-dimensions. IEEE Trans. Signal Process..

[97] Kim Y., Wang W., Carbonetto P., Stephens M. (2024). A flexible empirical bayes approach to multiple linear regression, and connections with penalized regression. J. Mach. Learn. Res..

[98] Jiao Q.Q., Yang X.J. (2024). Distributed variational measurement update for extended target tracking with random matrix. IEEE Trans. Aerosp. Electron. Syst..

[99] Yu W.C., Bondell H.D. (2023). Variational bayes for fast and accurate empirical likelihood inference. J. Am. Stat. Assoc..

[100] Liu X.L. (2022). Research on music genre recognition based on improved naive bayes algorithm. Mob. Inf. Syst..

[101] Yan W.X., Chen S.M., Lin D.Y., Wang S.Y. (2024). Variational bayesian-based generalized loss cubature kalman filter. IEEE Trans. Circuits Syst. II-Express Briefs.

